# Estimation of human trunk movements by wearable strain sensors and improvement of sensor’s placement on intelligent biomedical clothes

**DOI:** 10.1186/1475-925X-11-95

**Published:** 2012-12-14

**Authors:** Paolo Tormene, Michelangelo Bartolo, Alessandro M De Nunzio, Federica Fecchio, Silvana Quaglini, Cristina Tassorelli, Giorgio Sandrini

**Affiliations:** 1 Department of Computer Engineering and Systems Science, University of Pavia, Pavia, Italy; 2 Neurorehabilitation Unit, IRCCS Neurological Mediterranean Institute NEUROMED, Pozzilli (Isernia), Italy; 3 Department of Neurological Science, University of Pavia, Pavia, Italy; 4 Neurorehabilitation Unit, Neurological National Institute Casimiro Mondino Foundation, IRCCS, Pavia, Italy

**Keywords:** Wearable strain sensors, Trunk, Intelligent biomedical clothes, Rehabilitation

## Abstract

**Background:**

The aim of this study was to evaluate the concept of a wearable device and, specifically: 1) to design and implement analysis procedures to extract clinically relevant information from data recorded using the wearable system; 2) to evaluate the design and placement of the strain sensors.

**Methods:**

Different kinds of trunk movements performed by a healthy subject were acquired as a comprehensive data set of 639 multivariate time series and off-line analyzed. The space of multivariate signals recorded by the strain sensors was reduced by means of Principal Components Analysis, and compared with the univariate angles contemporaneously measured by an inertial sensor.

**Results:**

Very high correlation between the two kinds of signals showed the usefulness of the garment for the quantification of the movements’ range of motion that caused at least one strain sensor to lengthen or shorten accordingly. The repeatability of signals was also studied. The layout of a next garment prototype was designed, with additional strain sensors placed across the front and hips, able to monitor a wider set of trunk motor tasks.

**Conclusions:**

The proposed technologies and methods would offer a low-cost and unobtrusive approach to trunk motor rehabilitation.

## Background

In the evolution of neurorehabilitation techniques, the recovery of trunk function is assuming a significant level of interest as trunk stability is considered as an essential component of balance and its coordinated use with the extremities in daily functional activities [1,2]. Trunk muscles activity, and strength modulation, by means of appropriate neural control, was showed to be essential in trunk stability and limb movements [3]. Moreover, trunk muscles proprioceptive inputs play an important role in the control of gait [4,5] and could be taken into account for the interpretation of posture and movement problems [6] of patients suffering from balance disorders [7]. In functional rehabilitation, trunk control also emerged as a relevant factor in evaluation scales such as Activities of Daily Living and Sitting Balance Test, where it has been identified as a major predictor of motor and functional recovery [8,9].

Innovative technologies (robotics, virtual reality, wearable devices) have been proved to be helpful in neurorehabilitation, and their use for evaluation and recovery of motor functions, including trunk motions, appears promising [10,11]. In this context, wearable devices that monitor physiologic responses and interact with computer-based systems have the potential to increase recovery, as well as to promote personalized exercises and wellness regimens [12-17].

The increasing importance of the WT in several clinical applications including rehabilitative field, was well discussed by Bonato, 2009 and De Rossi et al., 2009, respectively [18,19].

The aim of this study was to describe an IBC designed for the recognition of trunk movements, that employs wearable strain sensors based on CE. This technology is not conceived to measure fine movements, but to recognize macro-movements, that are of interest in most trunk rehabilitation settings. Once validated, sensorized garments could be used in home-rehabilitation settings, with the possibility to automatically classify motor tasks, providing immediate feedback to the patient, and store motor performance for further remote control by therapist. The choice of this technology was motivated by two additional main aspects: the low cost, due to the quite simple industrial printing process, and the usability, since the garment was perceived like a common shirt.

Specifically, the paper described the design and implementation of analysis procedures to extract clinically relevant information from data recorded using the wearable system. Moreover, it described how to evaluate the design and placement of the strain sensors on the prototype, in order to devise improvements for the succeeding versions.

## Methods

### Recording system

CE are polymeric materials with piezoresistive properties, that can be smeared on fabrics by means of a cheap industrial printing process, without appreciably modifications in the mechanical properties of the underlying substrate [20]; they are non-toxic and waterproof. Even complex layouts of multiple sensors can be placed across normal elastic fabric surfaces, as close-fitting and elastic wearable clothes, across the specific body joints of which one wants to measure the movement.

**Figure 1:**
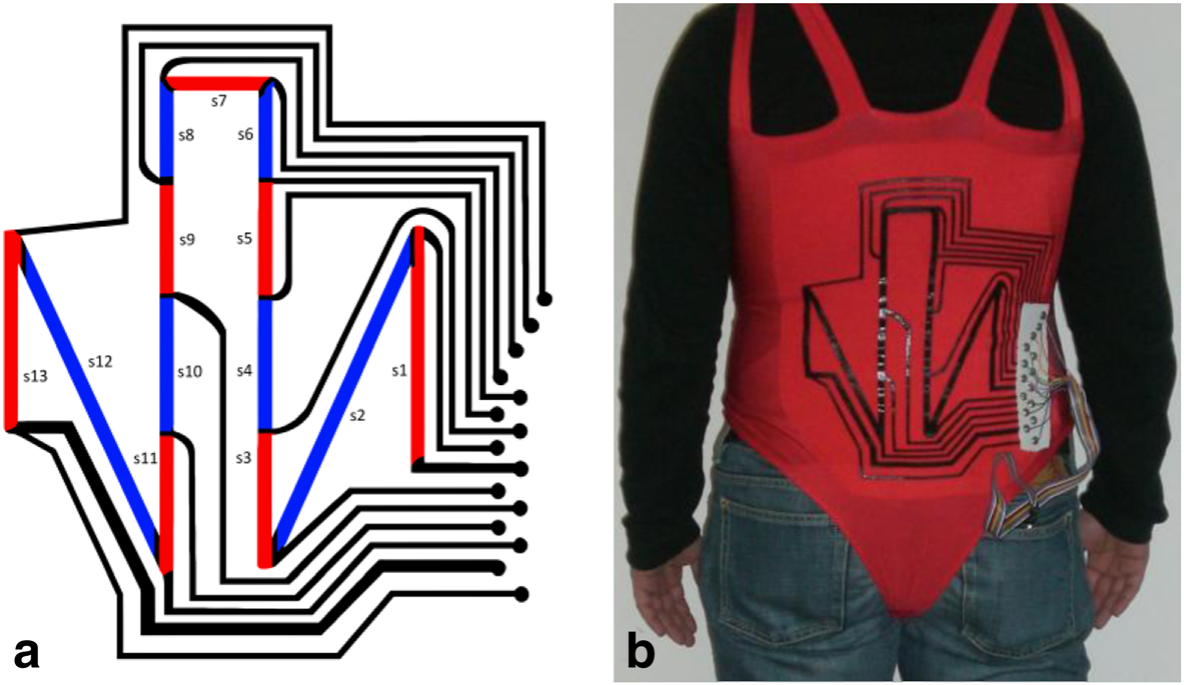
**Garment Prototype.a**) Layout of the placement of CE strain sensors on the garment prototype. Thick lines are sensors; thin lines are connection wires made of the same polymer. **b**) Picture of the garment from behind. The readout electronic device is placed in a pocket of the subject’s pants.

The prototype used in this study was designed to monitor trunk motions, so 13 CE strain sensors were realized across the back of a corset, Figure 1. A zipper in the front simplifies the wearing process; laces at the sides and Velcro straps allow to adapt the garment compensating for the different build of each subject, so sensors can be slightly pre-stretched, preventing them from wrinkling. A pocketsize electronic device supplies a direct current to the sensors (≈μA) and acquires the resistance offered by each of them. Signals are 32 Hz sampled **(**even higher than what’s necessary to monitor trunk flexions in a motor rehabilitation setting**),** digitized and sent via Bluetooth to the workstation that analyzes the data.

In order to evaluate the quality of the acquired signals, a previously assessed wireless inertial sensor, namely a MEMS**,** integrating a triaxial accelerometer and a triaxial magnetometer (whose data were processed by dedicated software, providing angles in degrees), was used as reference. During the recording sessions, the MEMS was placed (fixed by a double face band aid) on the spinous process C7 and used as a protractor, monitoring trunk bent; time stamped angles were 70 Hz sampled. Previous studies measuring body movements by means of MEMS [21], as well as preliminary assessments, indicate that the root mean square error of this class of device ranges approximately from 2 to 8 deg, i.e., an order of magnitude smaller than the required precision for our classification tasks.

### Testing protocol

A set of recording sessions was conducted, in order to collect a comprehensive set of 639 trunk movements. The movements were performed by a healthy subject varying speed and ROM. The subject gave written informed consent to participate in the study, which was performed according to the Declaration of Helsinki and the guidelines of the local ethics committee. Both the time series acquired by the sensorized garment and MEMS were collected in a data set, called *D* henceforth^a^. Each movement is uniquely identified by a set of indices (summarized in Table 1); namely, the generic element *D*_*sijlmn*_ is defined as follows: *s* is one of the five acquisition sessions, between each the subject doffed and donned the garment; *i* is one of the stages in which a session was articulated (the number of stages was variable, as documented in the dataset release notes; the subject made a 15 minutes pause between each stage, assuming a sitting position while resting); during each stage, the subject performed a series of motor exercises; *j* is one of the four types of exercises analyzed (flexion, extension, rightward or leftward bending); *l* indicates the range of motion of the movement (small, average**,** and large, corresponding to 30 deg, 60 deg**,** and 90 deg in case of flexion); *m* is the speed (qualitatively, slow, average, and fast) and *n* identifies different repetitions of a same movement. To identify a subset of *D* including multiple movements, we enclose the ranges for each index between parentheses; e.g., in *D*_*321(1-3)2(1-25)*_, *l* ranges between 1 and 3 and *n* between 1 and 25.

**Table 1:** **Description of the dataset indices**^**a**^

**Index of D**_**sijlmn**_	**Description**
s	acquisition session
	(the garment was doffed and donned before each session)
i	stage of a session
	(the subject assumed a sitting resting position for 15 minutes between each stage, without doffing the garment)
j	kind of trunk movement performed
	(flexion, extension, rightward or leftward bending)
l	Qualitative range of motion
	(small, average or large, e.g. 30, 60, 90 deg in case of flexion)
m	Qualitative speed (slow, average or fast)
n	Variable number of repetitions for each

### Data analysis and statistics

Measuring trunk flexion in the sagittal plane, the univariate angles measured by the inertial sensor and the 13-variate strains acquired by the CE sensors can be compared, formerly applying to the latter the PCA. PCA was suitable in this case to obtain dimensional reduction from 13 to a single principal component, and it was appropriate because we were not interested in absolute values, but in evaluating how the trends of the two kinds of sensors were correlated. In fact, the first principal component represents in a single dimension the redundant information measured by multiple sensors placed in the direction parallel to the spine.

In order to recognize the similarity between movements despite they were performed with different speeds, and a dynamic programming algorithm called DTW was used [22,23]. DTW performed non-linear deformations of the time axis to minimize the global multivariate Euclidean distance between the aligned time series. DTW returned a measure of dissimilarity (distance) between a couple of movements, so it was possible to compute the distribution of cross-distances between couples of time series selected from subsets of *D*. Thus, supervised classification could be done through a distance-based method such as Nearest-Neighbor. Namely, the current analyzed movement was recognized to be in the same class as the most similar movement in a training set where the class of each element was known. This analysis procedure allowed to automatically discriminate between different classes of movements, robustly with respect to execution speed, and it could be straightforwardly extended to a clinical recognition task with the aim to distinguish between normal and pathological actions.

To quantify the probability of our classifier random agreement (equal to 1 for classifiers that never fail any prediction, 0 for classifiers that perform as well as guess randomly), Cohen’s kappa-statistic measure was used.

## Results

**Figure 2:**
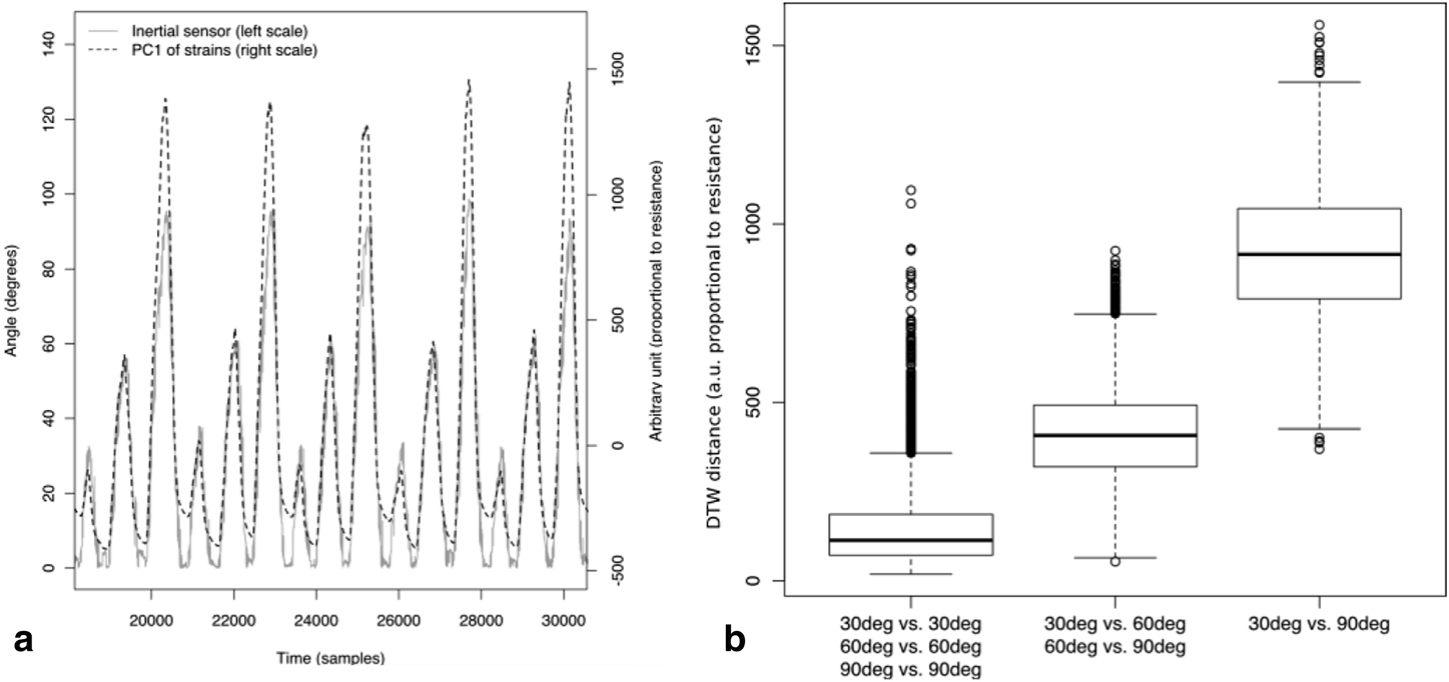
**Acquired data analysis.a**) The first principal component of the signals acquired by the 13 CE strain sensors is plotted together with the angle measured by the MEMS inertial sensor, versus time **b**) the boxplots represent distributions of “DTW distances” between couples of motor tasks, showing, as common, median, quartiles, largest and smallest observations, and outliers. Distances are computed between trunk flexions performed with: 1) the same ROM; 2) 30 degrees ROM difference; 3) 60 degrees difference.

Figure 2a shows signals acquired while the subject was repeating a series of trunk flexions with different ROM (*D*_*321(1-3)2(1-25)*_). Considering the whole set of trunk flexions, and confronting the signals acquired by the garment with those acquired by the MEMS, Spearman’s rank correlation coefficient calculated over the 126460 paired observations was equal to 0.88, with p < 0.001.

Considering the same subset *D*_*321(1-3)2(1-25)*_ as above, and computing the DTW distances between every pair of time series in the subset, the distribution *d* of distances was obtained. The three boxplots shown in Figure 2b represent three subsets of *d*, that are the distances between movements performed with the ROMs respectively indicated below each box. The boxplots are well separated, indicating that the DTW distance measure can be suitable to classify movements with respect to their ROM.

We analyzed three different classification tasks. The first one evaluated whether a small training set, containing only one example from each of the three defined classes (30 deg, 60 deg, 90 deg of trunk flexion), was sufficient to classify the remaining time series with acceptable accuracy. For each repetition of the experiment, we assumed as training set a different triplet of consecutive time series in the data subset *D*_*3(1-2)1(1-3)2(1-25)*_. The 136 experiments obtained were cross-validated and we computed the mean (.85) and standard deviation (.12) of the resulting Cohen's *kappa*. For the second task, the two stages of the session were taken separately, so the variability due to the 15 minutes pause between stages was neglectable (the count of experiments was in this case 134). Again, the results were cross-validated and the mean (.88) and standard deviation (.13) of *kappa* were computed. Finally, we analyzed the simplest classification task definable on the available data, leave-one-out cross-validation, attempting to classify each time series by means of a training set that contains all the others. In this case, every time series were correctly classified.

## Discussion

This study confirmed that trunk movements could be monitored and classified by means of sensorized garments employing the technology of wearable CE strain sensors. In fact, this garment can follow the angle of flexion in the sagittal plane as well as a MEMS inertial sensor.

**Figure 3:**
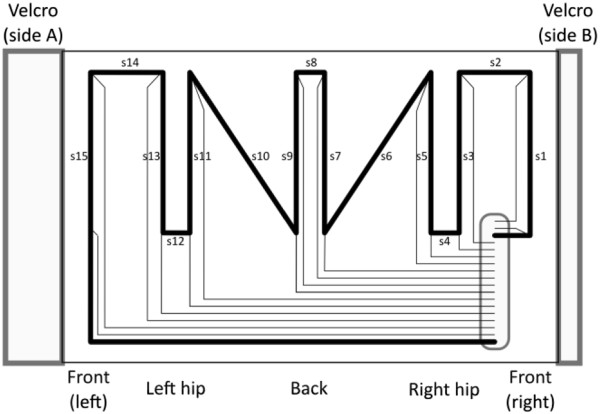
**New garment layout.** Draft of the layout of a new garment prototype. Note CE strain sensors surrounding the body, placed respectively: s1 and s15 in the front; s3, s5, s11 and s13 across the hips

Note that the sensors across the back were not suitable to monitor movements in other directions that would cause the CE stripes to wrinkle or to be deformed laterally during the action, instead of stretching parallel to their length. In order to properly monitor lateral bending and extension, for instance, we proposed the placement of additional sensors across the front and sides of the garment, as exemplified in the layout presented in Figure 3. Comparing the layouts presented in Figures 1 and 3, e.g., with respect to monitoring a trunk rightward bending, it was clear that in the former case most of the sensors were placed close to the spine and they did not significantly change their length during the movement, whereas in the latter case sensors s11 and s13 (placed across the left side) lengthened appreciably in accord to the bending action.

It is relevant to underline that this study represents a first step in the development of a device that will be employed to monitor patient’s motor activity, providing an automatic way to promptly detect cases of incorrect practice; such information, in principle, can be communicated remotely to a therapist who can set up a targeted intervention, if needed, and contemporarily store, quantify and document the results of the rehabilitation therapy session. The development of a device useful to acquire trunk movements, and provide feedback to patients about their performance, meanwhile enabling remote monitoring, would facilitate the rehabilitation activities in the home settings.

Although the proposed technologies offer an interesting approach to increase the intensity of trunk motor rehabilitation in neurological patients, the evaluation of the effectiveness of the device-assisted therapy still needs to be further addressed and it is beyond the scope of this work.

## Conclusions

Trunk movements can be acquired and examined with this new sensorized garment embedded with CE strain sensors. This device has not been developed for high precision trunk motions’ monitoring, but it represents an easy-to-use, inexpensive system for the wireless monitoring of the patient’s motor activity that poses a real advancement in the development of future useful and portable rehabilitating devices.

## Endnote

^a^The data, together with a detailed description, are available at the URL http://www.labmedinfo.org/projects/myheart/relatedResearch/.

## Abbreviations

WT: Wearable technology; IBC: Intelligent biomedical cloth; CE: Conductive elastomers; ROM: Range of motion; MEMS: Micro electro-mechanical system; PCA: Principal components analysis; DTW: Dynamic time warping.

## Competing interests

All the authors declare no financial or non-financial competing interests.

## Authors’ contributions

PT carried out the conception and design of the study, data acquisition, data interpretation, data analysis and drafted the manuscript. MB carried out the conception and design of the study, data acquisition, data interpretation and drafted the manuscript. AMDN carried out the interpretation of results, quality control and critical draft revision. FF carried out the data acquisition and data analysis. SQ carried out the conception and design of the study, data interpretation, and revised the manuscript. CT carried out the conception of the study and revised the manuscript. GS was the supervisor of research group, he acquired the funds, he carried out the conception and design of the study, data interpretation, and revised the manuscript. All authors have read and approved the final version of the manuscript.

## References

[B1] Dickstein R Shefi S Marcovitz E Villa Y Anticipatory postural adjustment in selected trunk muscles in post stroke hemiparetic patients Arch Phys Med Rehabil 2004 85 261 267 10.1016/j.apmr.2003.05.011 14966711

[B2] Bartolo M Serrao M Tassorelli C Don R Ranavolo A Draicchio F Pacchetti C Buscone S Perrotta A Furnari A Four-week trunk-specific rehabilitation treatment improves lateral trunk flexion in Parkinson's disease Mov Disord 2010 25 325 331 10.1002/mds.23007 20131386

[B3] Jacobs JV Henry SM Nagle KJ Low back pain associates with altered activity of the cerebral cortex prior to arm movements that require postural adjustment Clin Neurophysiol 2010 121 431 440 10.1016/j.clinph.2009.11.076 20071225 PMC2822008

[B4] Schmid M De Nunzio AM Schieppati M Trunk muscle proprioceptive input assists steering of locomotion Neurosci Lett 2005 384 127 132 10.1016/j.neulet.2005.04.059 15885899

[B5] Courtine G De Nunzio AM Schmid M Beretta MV Schieppati M Stance- and locomotion-dependent processing of vibration-induced proprioceptive inflow from multiple muscles in humans J Neurophysiol 2007 97 772 779 10.1152/jn.00764.2006 17065250

[B6] Adkin AL Bloem BR Allum JH Trunk sway measurements during stance and gait tasks in Parkinson's disease Gait Posture 2005 22 240 249 10.1016/j.gaitpost.2004.09.009 16278966

[B7] de Seze M Wiart L Bon-Saint-Come A Debelleix X de Seze M Joseph PA Mazaux JM Barat M Rehabilitation of postural disturbances of hemiplegic patients by using trunk control retraining during exploratory exercises Arch Phys Med Rehabil 2001 82 793 800 10.1053/apmr.2001.0820793 11387585

[B8] Wade DT Skilbeck CE Hewer RL Predicting Barthel ADL score at 6 months after an acute stroke Arch Phys Med Rehabil 1983 64 24 28 6849630

[B9] Kwakkel G Wagenaar RC Kollen BJ Lankhorst GJ Predicting disability in stroke–a critical review of the literature Age Ageing 1996 25 479 489 10.1093/ageing/25.6.479 9003886

[B10] Bartolo M Don R Ranavolo A Serrao M Sandrini G Kinematic and neurophysiological models: future applications in neurorehabilitation J Rehabil Med 2009 41 986 987 10.2340/16501977-0413 19841829

[B11] Ring H Technology in rehabilitation Eur Med Phys 2003 39 3 6

[B12] Giorgino T Tormene P Maggioni G Capozzi D Quaglini S Pistarini C Assessment of sensorized garments as a flexible support to self-administered post-stroke physical rehabilitation Eur J Phys Rehabil Med 2009 45 75 84 19293756

[B13] De Rossi D Veltink P Wearable technology for biomechanics: e-textile or micromechanical sensors? IEEE Eng Med Biol Mag 2010 29 37 43 20659856 10.1109/MEMB.2010.936555

[B14] Axisa F Schmitt PM Gehin C Delhomme G McAdams E Dittmar A Flexible technologies and smart clothing for citizen medicine, home healthcare, and disease prevention IEEE Trans Inf Technol Biomed 2005 9 325 336 10.1109/TITB.2005.854505 16167686

[B15] Chiari L Wearable systems with minimal set-up for monitoring and training of balance and mobility Conf Proc IEEE Eng Med Biol Soc 2011 2011 5828 5832 22255665 10.1109/IEMBS.2011.6091442

[B16] Preece SJ Kenney LP Major MJ Dias T Lay E Fernandes BT Automatic identification of gait events using an instrumented sock J Neuroeng Rehabil 2011 8 32 10.1186/1743-0003-8-32 21619570 PMC3113322

[B17] Giorgino T Lorussi F De Rossi D Quaglini S Posture classification via wearable strain sensors for neurological rehabilitation Conf Proc IEEE Eng Med Biol Soc 2006 1 6273 6276 17946755 10.1109/IEMBS.2006.260620

[B18] Bonato P Clinical applications of wearable technology Conf Proc IEEE Eng Med Biol Soc 2009 2009 6580 6583 19964699 10.1109/IEMBS.2009.5333997

[B19] De Rossi D Carpi F Lorussi F Scilingo EP Tognetti A Wearable kinesthetic systems and emerging technologies in actuation for upperlimb neurorehabilitation Conf Proc IEEE Eng Med Biol Soc 2009 2009 6830 6833 19964915 10.1109/IEMBS.2009.5334481

[B20] Lorussi F Scilingo EP Tesconi M Tognetti A De Rossi D Strain sensing fabric for hand posture and gesture monitoring IEEE Trans Inf Technol Biomed 2005 9 372 381 10.1109/TITB.2005.854510 16167691

[B21] Brennan A Zhang J Deluzio K Li Q Quantification of inertial sensor-based 3D joint angle measurement accuracy using an instrumented gimbal Gait Posture 2011 34 320 323 10.1016/j.gaitpost.2011.05.018 21715167

[B22] Tormene P Giorgino T Quaglini S Stefanelli M Matching incomplete time series with dynamic time warping: an algorithm and an application to post-stroke rehabilitation Artif Intell Med 2009 45 11 34 10.1016/j.artmed.2008.11.007 19111449

[B23] Giorgino T Tormene P Quaglini S A multivariate time-warping based classifier for gesture recognition with wearable strain sensors Conf Proc IEEE Eng Med Biol Soc 2007 2007 4903 4906 18003105 10.1109/IEMBS.2007.4353439

